# Chinese herbal experience for the 2019 novel coronavirus

**DOI:** 10.1186/s13054-020-03170-4

**Published:** 2020-07-21

**Authors:** Ronglin Jiang, Kungen Wang, Wei Mao, Wei Zhu, Weihang Hu, Liquan Huang

**Affiliations:** 1 Department of Intensive Care, The First Affiliated Hospital of Zhejiang Chinese Medical University, Hangzhou, 310006 Zhejiang People’s Republic of China; 2 Department of Intensive Care, Tianyou Hospital Affiliated to Wuhan University Of Science & Technology, Wuhan, HuBei People’s Republic of China; 3 Department of Intensive Care, Zhejiang Hospital, No. 12, Linyin Road, Hangzhou, 310000 Zhejiang People’s Republic of China

The novel coronavirus (COVID-19) has spread rapidly and become a severe global threat, with a reported acute respiratory distress syndrome (ARDS) incidence up to 40% [1]. According to a large survey, more than 14% patients were transferred to the intensive care unit care (ICU), and among those who received invasive mechanical ventilation, the mortality was as high as 88.1% [2].

Here we presented the data from a single ICU of Tianyou hospital in Wuhan, and according our experience, the overall mortality decreased in patients receiving Chinese herb therapy. From January 11, 2020, to March 17, 2020, a total of 37 patients confirmed with COVID-19 infection were admitted to ICU (Table 1), of whom seven patients were transferred to other hospitals and were excluded from this analysis. The general treatment regimens included glucocorticoids, antibiotics, hydroxychloroquine, and arbidol; however, the overall mortality rate remains as high as 78.1%. Chinese herb was applied in these patients since Feb 17. Thus, a total of nine patients received Chinese herbal therapy during the whole disease course (admitted to ICU after Feb 17), five patients received Chinese herbal therapy for a period of the whole disease course, and the rest fourteen patients had not received Chinese herbal therapy. Despite with limited sample size, the mortality rate decreased significantly after applying Chinese herbal to these patients (4/9 vs. 5/5 vs. 14/16, *p* = 0.033), especially in patients who received Chinese herbal therapy during the whole disease course. Further, these patients were also divided into two groups according to whether they had used Chinese herbal; a decreased trend of mortality was also observed (9/14 vs. 14/16, *p* = 0.134).

**Table 1:** Comparisons between survivors and non-survivors with coronavirus infection

Variables	All patients (*n* = 30)	Survivors (*n* = 7)	Non-survivors (*n* = 23)	*p*
Gender (male, %)	19 (63.3)	7 (100)	12 (52.1)	0.029
Age (years, %)	67.4 ± 9.2	65.7 ± 9.7	67.9 ± 9.2	0.582
PaO2/FiO2	107.7 ± 54.5	122.5 ± 52.3	102.2 ± 55.6	0.410
ICU length of stay	10.5 (5–14)	12 (10–12)	10 (4–15)	0.402
Intubation rate (%)	18 (60.0)	3 (42.8)	15 (65.2)	0.290
Comorbidities				
Hypertension (%)	10 (33.3)	3 (42.8)	7 (30.4)	0.657
Diabetes (%)	7 (23.3)	1 (14.2)	6 (26.0)	1.000
Use of Chinese herbal				0.033
The whole course (%)	9 (30)	5 (71.4)	4 (17.3)	
Part of the course (%)	5 (16.7)	0 (0)	5 (21.7)	
Not used (%)	16 (53.3)	2 (28.5)	14 (60.8)	

We understand our finding is unstable due to the limited sample size and potential cofounders. However, in China, Chinese herbal therapy has been fully applied to patients with COVID-19 infection in the middle stage of this epidemic and the effect is positive. The following content is the main Chinese herbal formulas for these nine patients:

- Formula 1: Xinren, Shigao, Gualuo, Dahuang, Mahuang, Tinglizi, Taoren, Caoguo, Binglang, Cangshu, Jinyinhua, Lianqiao, Hongjingtian.
- Formula 2: Fuzhi, Shengjiang, Huangqi, Renshen, Xinren, Shigao, Gualuo, Dahuang, Mahuang, Tinglizi, Taoren, Caoguo, Binglang, Cangshu, Gancao.

## Data Availability

Not applicable.
